# Caveolae-Specific CaMKII Signaling in the Regulation of Voltage-Dependent Calcium Channel and Cardiac Hypertrophy

**DOI:** 10.3389/fphys.2018.01081

**Published:** 2018-08-07

**Authors:** Shota Tanaka, Yasushi Fujio, Hiroyuki Nakayama

**Affiliations:** Laboratory of Clinical Science and Biomedicine, Graduate School of Pharmaceutical Sciences, Osaka University, Osaka, Japan

**Keywords:** caveolae, caveolin, CaMKII, L-type calcium channel, cardiac hypertrophy

## Abstract

Cardiac hypertrophy is a major risk for the progression of heart failure; however, the underlying molecular mechanisms contributing to this process remain elusive. The caveolae microdomain plays pivotal roles in various cellular processes such as lipid homeostasis, signal transduction, and endocytosis, and also serves as a signaling platform. Although the caveolae microdomain has been postulated to have a major contribution to the development of cardiac pathologies, including cardiac hypertrophy, recent evidence has placed this role into question. Lack of direct evidence and appropriate methods for determining activation of caveolae-specific signaling has thus far limited the ability to obtain a definite answer to the question. In this review, we focus on the potential physiological and pathological roles of the multifunctional kinase Ca^2+^/calmodulin-dependent kinase II and voltage-dependent L-type calcium channel in the caveolae, toward gaining a better understanding of the contribution of caveolae-based signaling in cardiac hypertrophy.

## Introduction

Caveolae are unique flask-like membrane invaginations of 50–80 nm in diameter, which are enriched in cholesterol and sphingolipids (Shaul and Anderson, 1998; Parton and Simons, 2007). Currently, caveolae are considered to be involved in various cellular functions such as lipid homeostasis, signal transduction, endocytosis, and transcytosis (Cheng and Nichols, 2016). The structure of caveolae is supported by two major component proteins: caveolins and cavins (Rothberg et al., 1992; Hill et al., 2008). Owing to their specific lipid composition, caveolae are highly concentrated in multiple signaling molecules, including receptors, kinases, and ion channels. Those include endothelial nitric oxide synthase (Garcia-Cardena et al., 1997), insulin (Nystrom et al., 1999), epidermal growth factor (Couet et al., 1997b), transforming growth factor-beta (Strippoli et al., 2015), P2X7 receptor (Gangadharan et al., 2015), and G-protein coupling signaling molecules (Insel et al., 2005). Thus, the caveolae are considered to function as a signaling platform to facilitate efficient and specific cellular responses against stress (Razani et al., 2002; Cohen et al., 2004; Harvey and Calaghan, 2012). However, determination of the specific physiological properties of caveolae signaling has been challenging due to the lack of efficient tools for direct visualization of kinase activation inside the caveolae. Understanding these signaling mechanisms of the caveolae can help to gain insight into their role in pathological mechanisms, particularly with regard to the contribution of cardiac hypertrophy, which remains controversial. Here, we review recent evidence on the signaling pathways and related molecules in the caveolae microdomain and their relation to cardiac pathogenesis, with a particular focus on Ca^2+^/calmodulin-dependent kinase II (CaMKII) and voltage-dependent L-type calcium channel (LTCC). This review can help to highlight targets of research focus and specific questions to tackle toward gaining a better understanding of the molecular mechanisms linking caveolae signaling and heart health, toward establishing new therapeutic strategies.

## Caveolae Microdomain and Signal Transduction

There are two types of structural proteins in caveolae: caveolins and cavins. Caveolin is comprised of three isoforms, caveolin-1, caveolin-2, and caveolin-3 (Rothberg et al., 1992; Scherer et al., 1996; Tang et al., 1996), with specific cellular distributions. For instance, caveolin-1 is dominantly expressed in endothelial cells, whereas caveolin-3 shows abundant expression in skeletal muscle cells and cardiomyocytes (Tang et al., 1996). These isoforms contain a common peptide sequence constituted by eight amino acids localized in the N-terminal cytosolic oligomerization domain (Tang et al., 1996). As a monomer, caveolin is comprised of three domains, oligomerization domain localized in N-terminus, caveolin scaffolding domain (CSD), and intramembrane domain in C-terminal part of the protein. Caveolin is inserted into the plasma membrane through intramembrane domain and CSD. Caveolin monomers assemble and form a oligomer, and contribute to caveolae formation (Sonnino and Prinetti, 2009). The CSD directly binds to a putative corresponding caveolin binding domain (CBD) identified in a number of signaling effectors localized in caveolae (Song et al., 1996; Nystrom et al., 1999; Kirkham et al., 2008; Taira et al., 2011). Couet et al. (1997a) identified a peptide sequence “RNVPPIFNDVYWIAF” as a CBD, which strongly binds to the CSD of caveolin 1 or caveolin 3. Currently, the physiological implication of the binding between CSD and CBD remains controversial (Collins et al., 2012). However, it is considered that CBD-fused protein binds to caveolin and demonstrates a specific localization in caveolae (Makarewich et al., 2012). Caveolin deficiency in a genetically engineered mouse model results in loss of the caveolae structure, indicating that caveolin is indispensable for the formation of caveolae (Park et al., 2002). Cavin contains four isoforms comprised of cavin-1 or polymerase I transcript factor (PTRF) (Hill et al., 2008), cavin-2 or serum deprivation protein response (SDPR) (Hansen et al., 2009), cavin-3 or SDR-related gene product that binds to C kinase (SRBC) (McMahon et al., 2009), and cavin-4 or muscle-related coiled-coiled protein (MURC) (Bastiani et al., 2009). Similar to caveolins, the cavin protein family shows a specific cellular distribution, and cavin-4 is thought to be a muscle-specific isoform. Cavin-1 is required for caveolae assembly and regulates the functions of caveolae by determining the localization of activated receptors (Li et al., 2014; Moon et al., 2014). In contrast, cavin-4 is dispensable for caveolae formation in cardiomyocytes, whereas it facilitates ERK1/2 recruitment to the caveolae and supports effective α1-andrenic receptor (AR) signaling activation in the development of cardiomyocyte hypertrophy (Ogata et al., 2014).

## Microdomain Signaling and Cardiac Hypertrophy

Cardiac hypertrophy is one of the predominant risks of heart failure (Lloyd-Jones et al., 2002). The development of cardiac hypertrophy is governed by multiple intracellular protein signaling cascades from the plasma membrane to nuclei (Heineke and Molkentin, 2006). Subcellular compartmentalization is considered to allow signaling-related proteins to carry out multiple biological functions using a relatively small number of membrane receptors. However, the precise contribution of microdomain signaling in cardiomyocyte hypertrophy remains elusive. Horikawa et al. (2011) reported that caveolin-3 overexpression in the mouse heart attenuates cardiac hypertrophy via upregulation of natriuretic peptide, suggesting the involvement of caveolin-dependent signaling in the development of myocyte hypertrophy. Balijepalli et al. (2006) reported that ARs and a component of the LTCC exist in caveolae microdomains. Ca^2+^-dependent signaling molecules such as calcineurin and CaMKII play vital roles in the development of cardiac hypertrophy, and activation of these molecules is associated with LTCC activity (Anderson et al., 2011; Chen et al., 2011). Makarewich et al. (2012) further demonstrated that caveolae-targeted inhibition of the LTCC mediates the attenuation of calcineurin activation induced by pacing stimulation without affecting Ca^2+^ influxes and transient in whole cells (Makarewich et al., 2012). To inhibit Ca^2+^ influxes in caveolae, they generated a fusion protein comprised of the caveolin-binding domain and Rem protein, which specifically inhibits LTCC activity. They found that caveolae-localized LTCCs are not involved in excitation-contraction coupling or the regulation of Ca^2+^, which governs contractility in isolated cardiomyocytes. However, the same group failed to demonstrate similar effects in the mouse heart in which pressure-overload was applied to a genetically engineered model expressing the fused Rem protein with the caveolin-binding domain (Correll et al., 2017). These results indicated that caveolae-related calcineurin/NFAT signaling alone is not sufficient for the development of cardiac hypertrophy.

## CaMKII in the Heart

CaMKII is a serine-threonine (Ser/Thr) kinase that is activated in a Ca^2+^/calmodulin-dependent manner. The activation of CaMKII is also regulated by autophosphorylation (Hudmon and Schulman, 2002), oxidation (Erickson et al., 2011), and glycosylation (Erickson et al., 2013). CaMKII phosphorylates a vast number of substrates such as ion channels, calcium handling proteins, and transcription factors (Anderson et al., 2011). Activation of CaMKII in the heart has been observed in both experimental models of cardiac hypertrophy and dysfunction as well as in patients suffering from heart failure (Zhang et al., 2003; Sossalla et al., 2010). Genetic ablation of dominant CaMKII isoforms in the heart attenuates cardiac hypertrophy or the transition to cardiac dysfunction after pressure overload (Zhang et al., 2003; Backs et al., 2009; Ling et al., 2009). Thus, CaMKII is considered to play a pivotal role in the development of cardiac hypertrophy and in the transition from the adaptive responses to heart failure (Swaminathan et al., 2012). In addition, the location of CaMKII activation is critical for its biological effects (Mishra et al., 2011). Two isoforms of CaMKII, CaMKIIδ, and CaMKIIγ, are mainly expressed in the heart, and the splicing isoform CaMKIIδ shows a unique subcellular localization. Such differential localization of CaMKII activation has been demonstrated to lead to a distinct intracellular function and cardiac phenotype (Zhang et al., 2002, 2003). Moreover, the cardiac overexpression of the cytosolic CaMKIIδ_C_ isoform in mice impairs excitation-contraction coupling (Zhang et al., 2003), whereas activation of the nuclear isoform CaMKIIδ_B_ mediates hypertrophic gene induction (Zhang et al., 2002). Further, the mitochondrial inhibition of CaMKII was shown to attenuate necrotic cell death (Joiner et al., 2012). Collectively, these findings indicate that the subcellular localization of CaMKII determines its biological effect based on the availability of substrate molecules, and is closely related to cardiac pathogenesis (Mishra et al., 2011). However, the specific biological role of CaMKII in the caveolae microdomain remains to be elucidated. In particular, deciphering its role in the membrane, as central platform for signal transduction, is required to gain a better understanding of its contribution to cardiac hypertrophy.

## Assessment of CaMKII Activation in the Caveolae Microdomain Using a Phosphor-Peptide Tag

One of the major obstacles in determining the precise pathophysiological role of CaMKII in the caveolae microdomain is the lack of efficient and simple methodology to assess microdomain-specific activation of the kinase. Conventionally, the activation of a kinase is determined biochemically by detecting the phosphorylation of its specific substrate using a radioisotope or fluorescence from a whole cell lysate. However, these methods are not suitable for the assessment of microdomain-specific signaling, since fraction preparation is complicated and time-consuming. Alternatively, the detection of phosphorylation using a phosphor-specific antibody is a simple, useful, and reliable method. The detection of mitogen-activated protein kinases (MAPKs) such as ERK and p38 MAPK is a representative example of the application of phosphor-specific antibodies for assessing signaling pathway activation.

**FIGURE 1:**
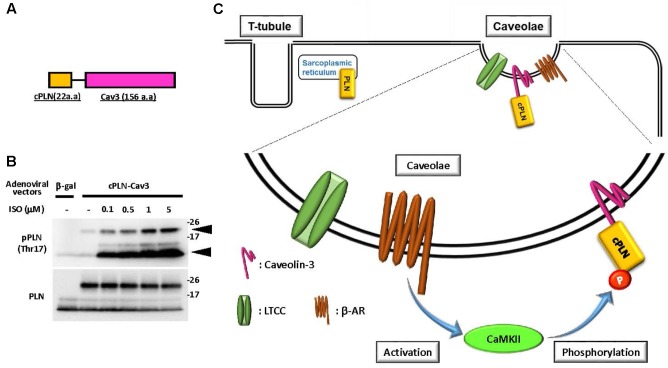
Caveolae-specific CaMKII activation was detectable using phosphor-peptide tags. **(A)** Schematic diagram of a fusion protein comprised of the cytosolic domain of phospholamban (cPLN) and caveolin-3 (Cav3). **(B)** Phosphorylation at threonine 17 of the cytosolic domain of phospholamban in the fusion protein and endogenous phospholamban. The fusion protein comprised of cPLN and caveolin-3 (cPLN-Cav3) was expressed by adenoviral gene transfer in neonatal rat cardiomyocytes (NRCMs). After 15 min of β-adrenergic stimulation with isoproterenol (ISO) at the indicated concentration, cells were harvested and their protein extracts were prepared. The phosphorylation of phospholamban at threonine 17 (Thr17), the CaMKII-specific phosphorylation site, was assessed using a phospho-specific antibody for Thr17 of PLN, and its level was enhanced in a dose-dependent manner in both cPLN-Cav3 (upper arrow) and endogenous PLN (lower arrow). The membrane was re-probed using an anti-PLN antibody. **(C)** Schema of the proposed method for caveolae-specific CaMKII activation using a fusion protein and phospho-specific antibody. CaMKII activation induced by β-adrenergic stimulation provokes phosphorylation of cPLN-Cav3, which is localized in caveolae.

We recently developed a novel tool to examine the caveolae-specific activation of CaMKII using a fusion protein comprising 22 amino acids of the cytosolic domain of phospholamban (PLN) fused to caveolin-3 (**Figure 1**) (Tonegawa et al., 2017). PLN is a 52-amino acid phospho-protein anchoring the membrane of the sarcoplasmic reticulum, which is comprised of a flexible cytosolic domain and an intramembrane domain (Tonegawa et al., 2017). The cytosolic domain of PLN contains two distinct phosphorylation sites: Ser16, which is mainly phosphorylated by cAMP-dependent kinase (PKA), and Thr17, which is specifically phosphorylated by CaMKII (Simmerman et al., 1986; Wegener et al., 1989; Hagemann and Xiao, 2002). Notably, each phosphorylation is detectable using the corresponding phospho-specific antibody, and the phosphorylation state is considered to represent activation of the corresponding kinases in the cytosol (Drago and Colyer, 1994). Therefore, we took advantage of these properties of PLN to develop a novel tool for determining the caveolae-specific activation of CaMKII. Indeed, phosphorylation of Thr17 in tagged cPLN localized in the caveolae was successfully detected using the phospho-specific antibody. Moreover, the phosphorylation level was enhanced by caveolae-specific activation or was suppressed by the caveolae-specific inhibition of CaMKII, indicating the reliability of this method (Tonegawa et al., 2017).

## LTCC and Cardiac Hypertrophy

The LTCC is a multi-protein complex composed of a pore-forming α-subunit and accessory subunits, including β-subunit proteins (Catterall, 2000). The β-subunits play important roles in regulation of channel activity as well as in channel membrane trafficking via interaction with the I-II intracellular loop of α-subunits (Catterall, 2000). Among the multiple splice variants of β-subunits, β2a is the dominant isoform in the heart. The LTCC serves as the primary source of Ca^2+^ influx for inducing contractions by triggering Ca^2+^-induced Ca^2+^ release (Bers, 2008). However, enhanced Ca^2+^ influxes caused by the targeted expression of the cardiac α1 or β2a subunit mediates or enhances cardiac hypertrophy (Muth et al., 2001; Chen et al., 2011). In addition, increased Ca^2+^ influxes caused by overexpression of the β2a subunit in feline cardiomyocytes or in the mouse heart driven by adenoviral expression or transgenesis induced pronounced myocardial Ca^2+^ overload that resulted in myocyte death (Chen et al., 2005; Nakayama et al., 2007). Therefore, functional sequestration of the LTCC subpopulation could be an important strategy to regulate cardiac pathogenesis. The LTCC has been shown to localize not only in the T-tubules but also in the plasma membrane microdomains such as the caveolae, and its localization is assumed to contribute to the distinct biological roles of the channel (Balijepalli et al., 2006; Best and Kamp, 2012; Shaw and Colecraft, 2013).

## CaMKII and LTCC in the Caveolae Microdomain Mediate Cardiac Hypertrophy

**FIGURE 2:**
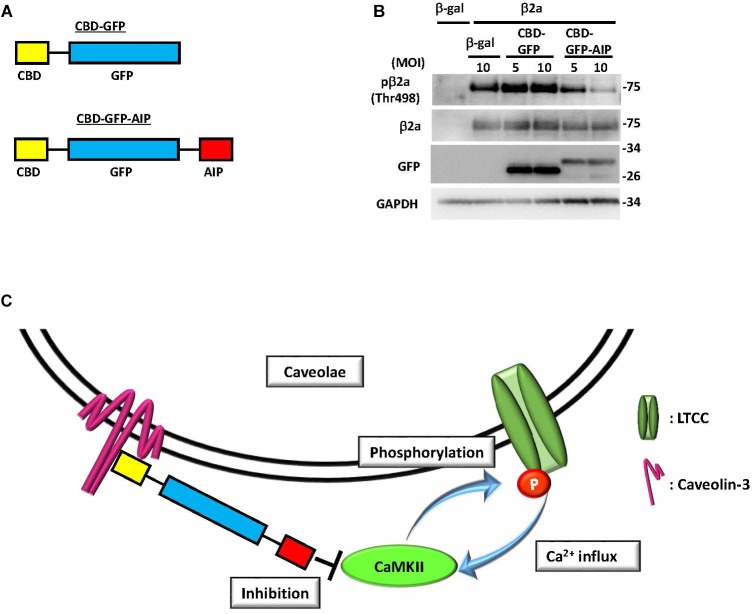
Inhibition of CaMKII specifically in caveolae abolished phosphorylation of the β2a subunit of the L-type calcium channel. **(A)** Schematic diagram of a fusion protein comprised of green fluorescent protein (GFP) tagged with caveolae-binding domain (CBD) and autocamtide-2-related inhibitory peptide (AIP), a CaMKII-specific inhibitory molecule. **(B)** Phosphorylation of the β2a subunit of the L-type calcium channel at threonine 498, a CaMKII phosphorylation site, in NRCMs expressing the β2a subunit or β-galactosidase (β-gal) as a control by adenoviral gene transfer. The additional adenoviral expression of a fusion protein, either CBD-GFP or CBD-GFP-AIP, was induced, and phosphorylation or expression levels were assessed by immunoblot analysis using the indicated antibodies. Phosphorylation of the overexpressed β2a subunit was substantially attenuated by CBD-GFP-AIP expression, indicating that phosphorylation of this protein occurs exclusively in caveolae. **(C)** Schema of the proposed mechanism based on immunoblot analysis. Phosphorylation of the β2a subunit by CaMKII induces a Ca^2+^ influx, which in turn elicits CaMKII activation to develop a positive feedback loop between the two molecules in the caveolae microdomain of NRCMs. Expression of CBD-GFP-AIP, which binds to caveolin-3, inhibits caveolae-specific CaMKII activation to terminate the positive feedback loop and abolish phosphorylation of the β2a subunit. MOI: multiplicity of infection. These figures were prepared with minor modifications from Tonegawa et al. (2017).

Besides cardiomyocytes, several reports suggested the involvement of CaMK in caveolae-related biological effects, such as 1α,25(OH)2D3-dependent signaling or P2X3 receptor-mediated Ca^2+^ influx (Chen et al., 2014; Doroudi et al., 2015). In cardiomyocytes, voltage-gated LTCC complex is a well-known substrate of CaMKII (Buraei and Yang, 2010). Phosphorylation of the β2 subunit by PKA or CaMKII has been proposed as an activation mechanism of LTCC mediated by extracellular stimuli (Bunemann et al., 1999; Koval et al., 2010). Several studies have also shown that the CaMKII- and PKA-mediated phosphorylation of the α1C subunit of LTCC facilitates its activity (Buraei and Yang, 2010; Weiss et al., 2013). However, the physiological importance of phosphorylation of the β2 subunit remains controversial. Mutant mice with a truncated β2 subunit lacking the phosphorylatable domain failed to show alteration of LTCC activity under physiological conditions (Brandmayr et al., 2012). By contrast, overexpression of a mutated β2 subunit resistant to CaMKII binding (L493A) and phosphorylation (T498A) resulted in attenuation of the cell death induced by delayed rapid-pacing (Koval et al., 2010). Therefore, upregulation of β2 subunit phosphorylation is thought to play a role in cardiac pathogenesis. In support of this hypothesis, increased expression of the LTCC β2a subunit and enhanced CaMKII activation are frequently observed in cases of human heart failure (Hullin et al., 2007; Anderson et al., 2011). Thus, sustained, excessive CaMKII activation is considered to be an upstream signaling event for increased LTCC opening probability, which is involved in excitation-contraction coupling dysfunction, myocardial hypertrophy, heart failure, and lethal arrhythmia (Wu et al., 1999; Rokita and Anderson, 2012; Zhu et al., 2016). However, whether the increased β2 subunit is phosphorylated by CaMKII and the subcellular location in which this critical event occurs remained unclear. Using our phospho-specific antibody, we demonstrated that the LTCC β2 subunit is phosphorylated by CaMKII in the caveolae to further induce CaMKII activation, possibly by increased Ca^2+^ influxes through the channel (**Figure 2**, Tonegawa et al., 2017). This suggested the possibility of a positive feedback loop between the β2 subunit and CaMKII that specifically occurs in the caveolae microdomain. This activation mechanism would contribute to the promotion of cardiac hypertrophy caused by chronic α1 adrenergic stimulation *in vivo,* since overexpression of the non-phosphorylated mutant of the β2a subunit failed to display enhancement of cardiac hypertrophy (Tonegawa et al., 2017). However, further investigation is required to clarify the direct link between caveolae-specific CaMKII signaling and cardiac hypertrophy *in vivo*, using genetically engineered mouse models such as caveolae-specific expression of constitutive active CaMKII (activation) or a CaMKII-specific inhibitory peptide (inhibition).

## Conclusion and Prospects

We have here summarized the current knowledge on the effects of caveolae-specific signal activation in relation to the pathogenesis of cardiac hypertrophy. The related molecules such as CaMKII and LTCC have multiple cellular functions that seem to depend on the corresponding subcellular localization of the molecules, including intracellular organelles and microdomains. Thus, regulation of location-dependent signal activation is a potential therapeutic target for heart failure. For instance, inhibition of the specific population of CaMKII or LTCC, those which are involved in the development of cardiac hypertrophy or induction of cell death, could potentially improve the prognosis of patients with heart failure without disturbance of excitation-contraction coupling. However, several questions remain to be answered regarding the role of microdomain-specific signaling in the development of cardiac hypertrophy and heart failure. First, the contribution of other microdomains such as lipid rafts (Dodelet-Devillers et al., 2009) or couplons (Chopra and Knollmann, 2013) needs to be determined, which requires the development of novel and simple tools to assess these microdomain signals. Second, the physiological relevance of the regulation of these microdomain-specific signaling pathways should be determined *in vivo*. Third, the methodology for assessing signals other than CaMKII needs to be developed. Finally, the role of the specific activation of these signals in subcellular organelles such as the mitochondrion should be determined. Methods based on a phosphorylatable peptide-tag have great potential to help tackle these questions. Taken together, the evidence accumulated to date indicates that selective inhibition of target molecules involved in caveolae-specific signaling based on their subcellular location could be a promising therapeutic tool to treat cardiac hypertrophy and heart failure in the future.

## Author Contributions

ST and HN wrote the manuscript. YF checked and approved the manuscript.

## Conflict of Interest Statement

The authors declare that the research was conducted in the absence of any commercial or financial relationships that could be construed as a potential conflict of interest.
